# Differences in age-related effects on brain volume in Down syndrome as compared to Williams syndrome and typical development

**DOI:** 10.1186/1866-1955-6-8

**Published:** 2014-04-09

**Authors:** Mary Ellen I Koran, Timothy J Hohman, Courtney M Edwards, Jennifer N Vega, Jennifer R Pryweller, Laura E Slosky, Genea Crockett, Lynette Villa de Rey, Shashwath A Meda, Nathan Dankner, Suzanne N Avery, Jennifer U Blackford, Elisabeth M Dykens, Tricia A Thornton-Wells

**Affiliations:** 1 Center for Human Genetics and Research, Department of Molecular Physiology & Biophysics, Vanderbilt University School of Medicine, 37232-0700, 519 Light Hall, Nashville, TN, USA; 2 Medical Scientist Training Program, Vanderbilt University School of Medicine, Nashville, TN, USA; 3 Short-Term Training Program Undergraduate Research Fellow, Vanderbilt University, Nashville, TN, USA; 4 Neuroscience Graduate Program, Vanderbilt University, Nashville, TN, USA; 5 Interdisciplinary Studies in Neuroimaging of Neurodevelopmental Disorders, The Graduate School, Vanderbilt University, Nashville, USA; 6 Graduate Program in Clinical Psychological Sciences, Department of Psychology, Vanderbilt University, Nashville, TN, USA; 7 Department of Psychiatry, Vanderbilt University School of Medicine, Nashville, TN, USA; 8 Vanderbilt Kennedy Center for Research on Human Development, Vanderbilt University, Nashville, TN, USA; 9 Department of Psychology and Human Development, Vanderbilt University, Nashville, TN, USA; 10 Vanderbilt University Institute of Imaging Science, Vanderbilt University, Nashville, TN, USA

**Keywords:** Down syndrome, Williams syndrome, Neurodevelopmental disorder, Brain volume, *APOE*, MRI, Accelerated aging, Neuroimaging genetics, Alzheimer’s disease

## Abstract

**Background:**

Individuals with Down Syndrome (DS) are reported to experience early onset of brain aging. However, it is not well understood how pre-existing neurodevelopmental effects versus neurodegenerative processes might be contributing to the observed pattern of brain atrophy in younger adults with DS. The aims of the current study were to: (1) to confirm previous findings of age-related changes in DS compared to adults with typical development (TD), (2) to test for an effect of these age-related changes in a second neurodevelopmental disorder, Williams syndrome (WS), and (3) to identify a pattern of regional age-related effects that are unique to DS.

**Methods:**

High-resolution T1-weighted MRI of the brains of subjects with DS, WS, and TD controls were segmented, and estimates of regional brain volume were derived using FreeSurfer. A general linear model was employed to test for age-related effects on volume between groups. Secondary analyses in the DS group explored the relationship between brain volume and neuropsychological tests and *APOE*.

**Results:**

Consistent with previous findings, the DS group showed significantly greater age-related effects relative to TD controls in total gray matter and in regions of the orbitofrontal cortex and the parietal cortex. Individuals with DS also showed significantly greater age-related effects on volume of the left and right inferior lateral ventricles (LILV and RILV, respectively). There were no significant differences in age-related effects on volume when comparing the WS and TD groups. In the DS group, cognitive tests scores measuring signs of dementia and *APOE* ϵ4 carrier status were associated with LILV and RILV volume.

**Conclusions:**

Individuals with DS demonstrated a unique pattern of age-related effects on gray matter and ventricular volume, the latter of which was associated with dementia rating scores in the DS group. Results may indicate that early onset of brain aging in DS is primarily due to DS-specific neurodegenerative processes, as opposed to general atypical neurodevelopment.

## Background

It has been suggested that individuals with Down syndrome (DS) experience early onset, or perhaps accelerated, brain aging as evidenced by significant age-related reduction in brain volume, but to date, it is not known whether this is an effect of the accumulated neurotoxic pathology evident in DS or a pre-existing feature of atypical neurodevelopment [1]. In the current study, we aimed to dissociate the contribution of neurodevelopment versus neurodegeneration in regional brain volume in DS. We compared a wide age range of adults with DS to adults with a different neurodevelopmental disorder, Williams syndrome (WS), and we also compared both neurodevelopmental disorders to a typically developing (TD) control group.

### Etiology and presentation of Down syndrome and Williams syndrome

DS is a neurodevelopmental disorder caused by the presence of three copies of chromosome 21 (trisomy 21). It occurs in one in every 691 live births in the US [2] and is the most common genetic cause of intellectual disability [3,4]. The degree of cognitive impairment associated with DS ranges from mild to severe, with the mean IQ of 50, or moderate intellectual disability [4]. Individuals with DS exhibit deficits in language, verbal short-term memory, and explicit long-term memory; whereas visuospatial short-term memory, associative learning, and implicit memory are relatively preserved [5]. Advances in the treatment of medical comorbidities, such as heart defects and digestive malformations, have resulted in dramatic improvements in life expectancy for individuals with DS living in the US, rising from nine years in the early-twentieth century [6] to nearly 60 years in 2010 [7]. Although individuals with DS present with a unique cognitive and behavioral profile, they do share some basic characteristics with individuals who have WS.

WS is a neurodevelopmental disorder caused by the hemizygous deletion of 26 to 28 genes on chromosome 7 [8]. The prevalence of WS is one in every 7,500 live births [9]. As with DS, WS is associated with intellectual disability. The average IQ for individuals with WS is approximately 50 to 60, indicating mild to moderate intellectual disability [10,11]. The WS cognitive profile is characterized by deficits in visuospatial and implicit memory as well as strengths in language, verbal short-term memory, face and object recognition, and music processing skills [12-16]. In addition, individuals with WS often demonstrate increased non-social anxiety and phobias, paired with hypersociability and heightened empathy [17-19]. Similar to individuals with DS, persons with WS have experienced a significant increase in mean life expectancy following advances in treatment for medical comorbidities, particularly cardiac defects. There is very little literature on aging or life expectancy in WS, but there are documented cases of persons with WS who lived to be 70 years old [20].

### Brain morphometry in Down syndrome and Williams syndrome

Children and young adults with DS or WS have an overall smaller brain volume compared to TD individuals of similar age [21]; however the specific brain areas that show significant volumetric differences compared to TD are distinct for each of the neurodevelopmental disorders. Individuals with DS have smaller frontal, amygdalar, and cerebellar volumes compared to TDs; whereas individuals with WS have smaller midbrain, thalamic, basal ganglia, and occipital and superior parietal lobe volumes compared to age-matched TDs [22,23]. It is important to also note that individuals with DS have an increase in parahippocampal volume and relatively preserved lenticular nuclei, basal ganglia, and occipital lobe volumes [4,5,24]. Young adults with WS have relatively preserved frontal lobe, anterior cingulate, superior temporal and fusiform gyrus, amygdalar, and cerebellar volumes compared to TDs [5,22,24].

In addition to the pre-existing smaller volumes of frontal, amygdalar and cerebellar structures, older adults with DS (>50 years of age) have been shown to also have smaller whole prefrontal, posterior cingulate, hippocampal, and parahippocampal volumes when compared to age-matched TD adults [5]. Studies have shown that as individuals with DS age, they exhibit a similar pattern of neurodegeneration to that seen in the early stages of Alzheimer’s disease in the general population, in which the earliest neuropathological changes present in the medial-temporal lobe and progress to neocortex and subcortical regions [25]. However, in individuals with DS these neuropathological changes occur at a much younger age compared to the general population, which has been attributed to early onset, or perhaps accelerated, brain aging [1].

At present, very few studies have assessed changes in brain morphology in older adults with WS [26]. Studies have shown an overall 15% smaller brain volume in adults with WS between 19 and 52 years of age compared to age-matched TD controls [20,21]; however, they found no difference in the magnitude of this finding between older individuals with WS and a group of younger persons with WS, suggesting the effect might not be age-related [20].

### Study aims

The aims of the current study were to: (1) confirm previous findings of age-related brain changes in DS versus TD, (2) document any age-related differences in brain volume seen in WS versus TD, and (3) test for age-related effects that are unique to DS. If the changes seen in DS are primarily due to DS-specific neurodegenerative processes, then we would hypothesize that in the DS group, the age-related effects would be greater than those in the TD and WS groups. If, however, these changes are instead associated with a non-specific vulnerability due to atypical neurodevelopment, then we would hypothesize that both DS and WS groups would show greater age-related differences as compared to TD adults than they would compared to each other.

## Methods

### Study participants

The current study included 14 DS adults (7 males; mean age 39; age range: 19 to 63), 41 WS adults (24 males; mean age 26, age range: 16 to 58), and 82 TD adults (40 males; mean age 36, age range: 18 to 90). Adults with DS or TD were recruited using flyers and website postings with Institutional Review Board-approved language targeting adults over 18 years of age. For adults with DS, we further recruited from local and regional educational centers for individuals with intellectual disabilities, community-based assisted living centers, caregiver support groups, and employment assistance programs. Participants with WS were recruited through the annual Academy of Country Music Lifting Lives Music Camp, which is organized by the Vanderbilt Kennedy Center for Research on Human Development. All participants with WS or DS exhibited the physical, cognitive, and behavioral profile of WS and DS, respectively, and they previously had received a clinical diagnosis of the disease. Adults with typical neurodevelopment were ascertained either as age-matched controls for study participants with WS or as healthy older adults who served as controls for a general population study of age-related cognitive impairment. The three groups were tested for differences in age and sex, using an independent samples *t*-test and a chi-square test, respectively. Demographic characteristics along with their corresponding statistical values are detailed in Table 1.

**Table 1:** Sample demographics for participants with Down syndrome (DS), Williams syndrome (WS), and typically developing (TD) controls

										**Group contrasts**					
	**DS (N = 14)**			**TD (N = 82)**			**WS (N = 41)**					**DS versus WS versus TD**			
	**N**	**%**		**N**	**%**		**N**	**%**				**χ2**	***P*-value**		
**Male sex**	7	50		40	49		24	59				1.89	0.39		
										**DS versus TD**		**WS versus TD**		**DS versus WS**	
	**Range**	**Mean**	**SD**	**Range**	**Mean**	**SD**	**Range**	**Mean**	**SD**	***t***	***P*-value**	***t***	***P*-value**	***t***	***P*-value**
**Age (years)**	19 to 63	39	13	18 to 90	36	18	16 to 58	26	8	0.61	0.54	-3.44	1.0E-03	4.31	7.1E-05
	**Range**	**Median**	**SD**												
**DLD-SCS**	0 to 21	0.5	8												
**DLD-SOS**	0 to 18	5	5												

SD = standard deviation, N = number or count, % = percentage of subjects in the group corresponding to the preceding count, *t* = *t*-statistic, and χ^2^ = chi-square test of independence statistic.

Participants with TD and caregivers of individuals with DS or WS gave informed consent, while participants with DS or WS gave informed assent for this study. All study procedures were approved by the Vanderbilt University Institutional Review Board.

### Magnetic resonance imaging acquisition

Adults participated in a magnetic resonance imaging (MRI) scan in a Philips Achieva 3-Tesla scanner (Philips Medical Systems, Inc., Best, Netherlands) using an eight-channel SENSE head coil, housed in the Vanderbilt University Institute of Imaging Science (Nashville, TN, USA). High-resolution three-dimensional anatomical T1-weighted MRI images were acquired using a turbo field echo sequence with full brain coverage and the following parameters: field of view = 256 × 256 mm^2^; in plane voxel resolution = 1 × 1 mm^2^; repetition time = 8.9 ms; echo time = 4.6 ms; flip angle = 8°; slice thickness = 1 mm and 170 slices with no slice gap.

### Neuroimaging analysis

To parcellate the brain into cortical and subcortical tissue classes and derive quantitative estimates of brain volume, we used an automated, non-biased atlas-based Bayesian segmentation procedure, applied in FreeSurfer v.5.0 (http://surfer.nmr.mgh.harvard.edu/) [27]. FreeSurfer preprocessing for volumetric T1-weighted images included: brain extraction and removal of non-brain tissue using a hybrid watershed/surface deformation procedure [28]; automated spatial transformation and white matter segmentation of subcortical volumetric structures [29]; intensity normalization, tessellation of gray matter/white matter boundary and automated topology correction [30]; and surface deformation following intensity gradients to optimally place gray matter/white matter and gray matter/cerebrospinal fluid borders at the location where the greatest shift in intensity defines the transition to the other tissue class [27]. Image outputs from each stage of FreeSurfer processing were visually inspected independently by three imaging analysts (CME, MEK). Only images that passed quality control by both analysts were used; seven DS, five WS, and five TD adults were not included in the analysis because of failure to pass quality control, leaving a total of 14, 58 and 81 adults in each category, respectively. Quantitative estimates of volume were derived in a large set of spatially distinct region of interests (ROIs) that covered the entire brain, as specified in the Desikan atlas [31]. This atlas includes parcellations of gray and white matter and segmentations of subcortical gray matter, and also includes summary volumes (that is total cortex volume). Parcellations of the gray and white matter and segmentations of subcortical gray matter were included, along with two summary measurements of total gray and total white matter (see Additional file 1: Tables S1a and b for complete list of ROIs included and those excluded, respectively). Total intracranial volume (ICV) was also estimated in FreeSurfer, and all ROI measures were normalized to ICV for subsequent analyses.

### Genotyping

Adults with DS had blood drawn for DNA, which was directly genotyped for *APOE* using pre-made TaqMan SNP genotyping assays from Applied Biosystems (ABI; Foster City, CA, USA). Negative controls (no template) and positive controls (DNA samples with known genotypes from Coriell Institute for Medical Research, Camden, NJ, USA) were included on the plate for assay validation. Since genotyping was performed in a research laboratory that is not CLIA-certified, genotyping results were not returned to patients or their clinicians.

### Cognitive testing

For all participants with Down syndrome, we conducted a comprehensive battery of cognitive and neuropsychological tests (see Additional file 1: Table S2), including the Dementia Questionnaire for People with Learning Disabilities (DLD; Harcourt Assessment, Amsterdam, Netherlands, 2006) [32]. Although there is no ‘gold standard’ for assessing dementia in individuals with DS, studies have shown that the DLD is useful in the differential diagnosis of dementia [33,34]. The DLD is a 50-item questionnaire that consists of eight subtests (short-term memory, long-term memory, and spatial and temporal orientation, speech, practical skills, mood, activity and interest, and behavioral disturbance) that assess both cognition and social skills. For each item, a score of 0 indicates no deficit, 1 indicates moderate deficit, and 2 indicates severe deficit. The sum of cognitive scores (SCS) is calculated from the short-term memory, long-term memory, spatial and temporal orientation subtests, and the range of possible scores for the DLD-SCS is 0 to 44. The sum of social scores (SOS) is calculated from the speech, practical skills, mood, activity, and interest and behavioral disturbance subtests, and the range of possible scores for the DLD-SOS is 0 to 60. Higher scores on each subtest indicate greater impairment. A masters-level study coordinator with training and experience in cognitive and neuropsychological assessment administered the DLD. Complete results of neuropsychological testing for the DS participants, as well as *APOE* ϵ4 carrier status, can be seen in Additional file 1: Table S2. We also note that none of the participants with DS were taking anti-depressants, anti-psychotics or cholinesterase-inhibitors, which could confound cognitive testing results, although one person was taking the anti-convulsant ‘Lamictal,’ which is a sodium channel blocker.

### Statistical analysis

Our first two aims were to (1) confirm previous findings of early age-related changes in DS compared to TD and (2) test for a comparable effect in WS relative to TD. In order to approach these aims, we implemented a general linear model in R (http://www.R-project.org) across 103 separate regions of interest:

$$\begin{array}{l} \left(\mathrm{ICV}‒\mathrm{corrected}\;\mathrm{volume}\;\mathrm{of}\;\mathrm{each}\;\mathrm{ROI}=\right)\\ \;\left(\mathrm{Group}+\mathrm{Age}+\mathrm{Sex}+\left(\mathrm{Group}\;\times \;\mathrm{Age}\right)\right) \end{array}$$

Group status was dummy-coded in the regression model with TD set as the reference category. Thus, our model included two group main effects (DS as 0 or 1 and WS as 0 or 1) and two interaction terms: age × DS and age × WS which statistically compare the age-related slopes of regional volume between the respective diagnostic category and TD controls. This method and a three category method in ANCOVA are statistically equivalent [35]. However, in the linear regression method used here, the *t*-tests based on dummy-coded variables directly test the alternative hypothesis that the coded group differs from the reference group (in this case, TD controls), which aids in the interpretation of results.

Sex was coded as a binary discrete variable (male as 0; female as 1). A Bonferroni corrected significance threshold of *P* < 4.85 × 10^-4^ was applied to the interaction terms in order to correct for the 103 ROIs tested (see Additional file 1: Table S2a for list of ROIs tested). A *post hoc* analysis was conducted to determine the sensitivity of results due to outliers. Outliers were defined as adults with ICV-corrected total gray matter volume outside of the grand mean ± two standard deviations (calculated in SPSS, http://www.ibm.com/software/analytics/spss/).

A final aim was to test for age-related effects unique to DS. The same general linear model was used (ICV-corrected volume of each ROI = Group + Age + Sex + (Group × Age)), but we only compared DS and WS subjects (WS = 0 and DS = 1). A Bonferroni correction was applied to determine the significant interaction terms, and again a *post hoc* analysis was performed after removing statistical outliers.

### Secondary exploratory analyses

In order to further explore age-related volume that is specific to DS, we performed linear regression to test for an association between brain volume and other cognitive and genetic risk factors. Since DLD and genotyping data were only collected for participants with DS, analyses were restricted to the DS group. To control for Type I error, we restricted our analysis to brain regions that showed significant age-related effects, which were stronger in DS relative to TD and WS. Predictors included age, sex, and the variable of interest. For each ROI, we tested for an association with DLD-SOS or DLD-SCS as the continuous variable of interest. We report the *t*-statistic for the variable of interest (DLD-SOS or DLD-SCS), along with its unadjusted *P*-value, and we report the change in R^2^ comparing the full model with the variable of interest to the reduced model with only age and sex.

Next we performed an exploratory analysis to determine the relationship between *APOE* ϵ4 carrier status and age-related volume effects in DS. The absence/presence of *APOE* ϵ4 alleles was coded as 0/1, respectively. We report the unadjusted *P*-value of the *APOE* term and the change in R^2^ for the full model with *APOE* to the reduced model with only age and sex as predictors.

## Results

**Figure 1:**
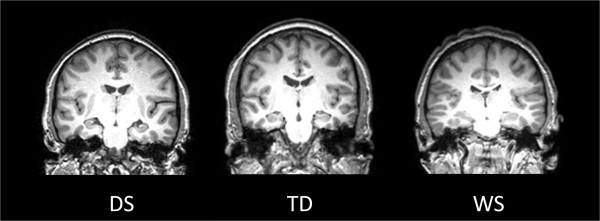
**Representative T1-weighted MRI for each group (Down syndrome (DS), typically developing (TD), and Williams syndrome (WS)).** The subjects are 35, 36, and 38 years old respectively. The inferior lateral ventricles can be seen as hypo-intense spaces around the hippocampus in the temporal lobes.

**Figure 2:**
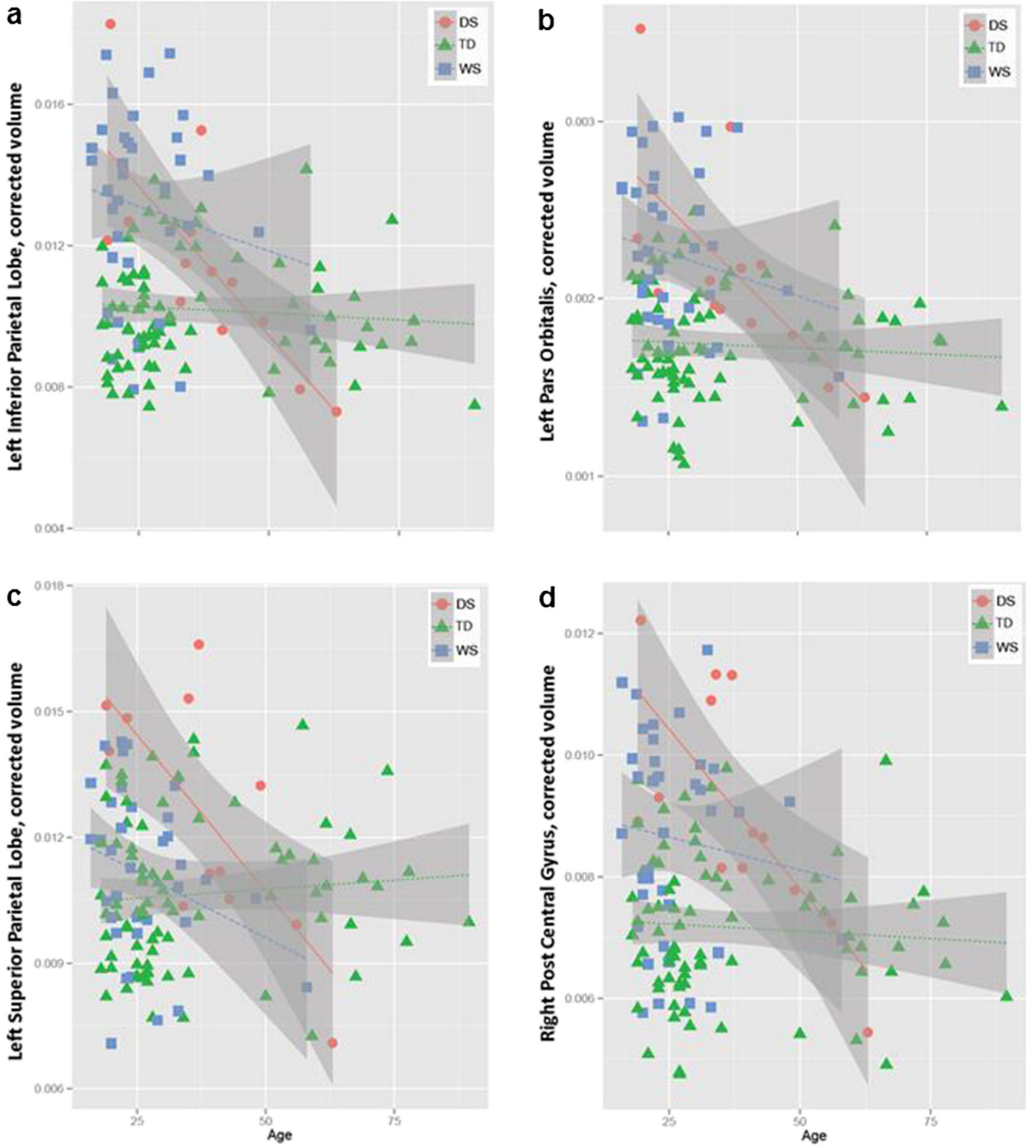
Regional brain volumes normalized to total intracranial volume (ICV) are plotted in relationship to age across the three subject groups (Down syndrome (DS) in red, typical development (TD) in green, and William syndrome (WS) in blue) in (a) left inferior parietal lobe, (b) left pars orbitalis, (c) left superior parietal lobe, and (d) right post central gyrus.

**Figure 3:**
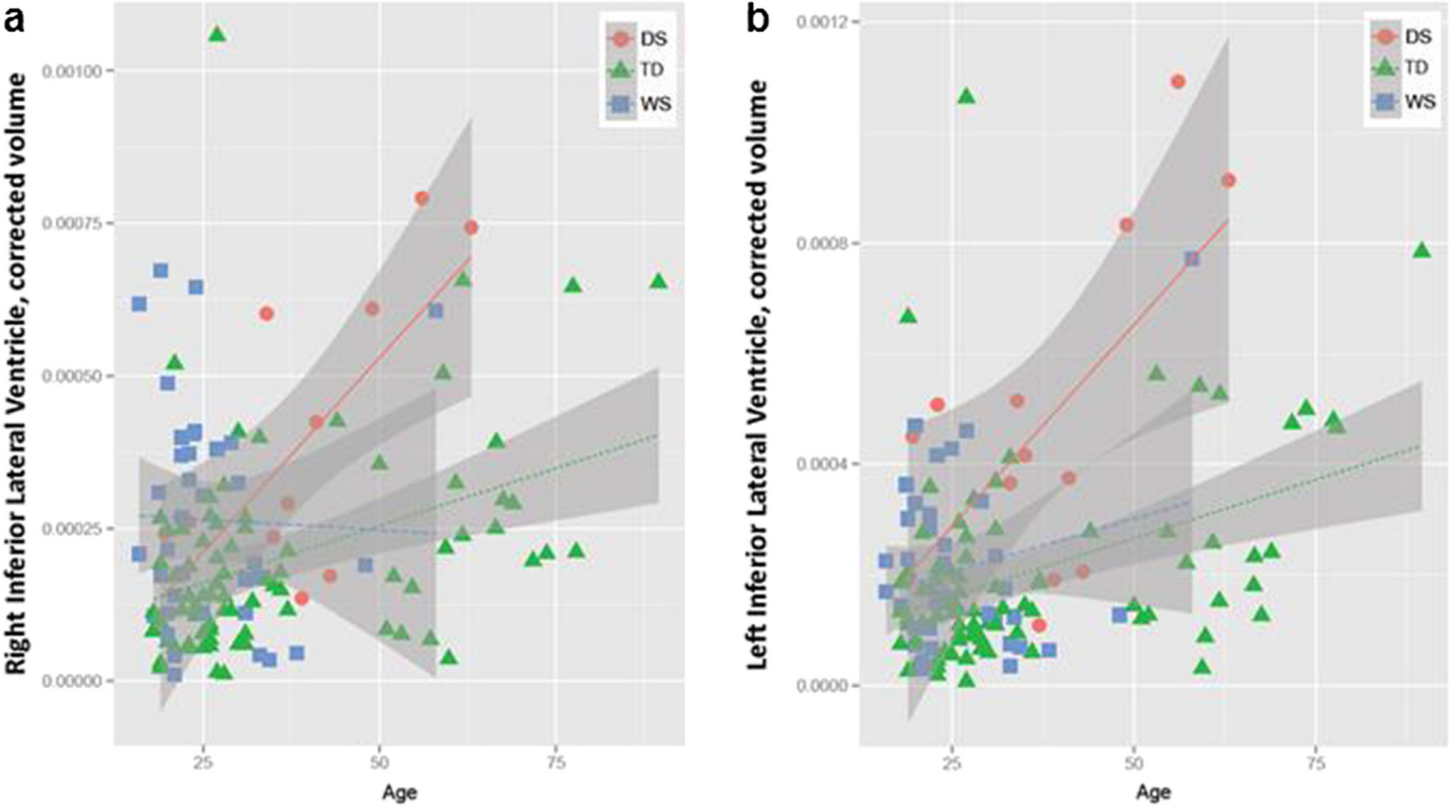
Regional brain volumes normalized to total intracranial volume (ICV) are plotted in relationship to age across the three subject groups (Down syndrome (DS) in red, typical development (TD) in green, and William syndrome (WS) in blue) in: (a) left inferior lateral ventricle (LILV) and (b) right inferior lateral ventricle (RILV).

Example T1-weighted MR images for each group can be seen in Figure 1. Our first analysis aimed to (1) replicate previous findings of age-related effects on brain in DS and (2) test for similar effects in WS. Consistent with previous findings, the DS group showed significantly greater age-related effects on gray matter volume relative to TD controls in the regions of the orbitofrontal cortex (the left pars orbitalis) and the parietal cortex (the left superior parietal lobe, the left inferior parietal lobe and the right post central gyrus; Table 2, Additional file 1: Table S3; Figure 2). Individuals with DS also showed significant age-related effects on volume of the left and right inferior lateral ventricles (LILV and RILV, respectively, Figure 3). In contrast, there were no significant differences in age-related volume between WS and TD controls. The summary measurements of total white matter and total gray matter volumes were also analyzed, and the DS group showed greater age-related effects on total gray matter volume (unadjusted *P* = .007) while there was no significant effect in the total white matter volume measure (*P* = .055).

**Table 2:** Brain regions where the relationship between age and volume was significantly different between participants with Down syndrome (DS) versus typical development (TD)

**Region of interest**	**Group (DS versus TD) × Age Interaction**		
	***t***	***P*-value**	**Difference in R**^**2**^
LILV	4.31	3.25E-05	0.09
RILV	4.05	9.03E-05	0.10
Left superior parietal	-4.02	1.01E-04	0.08
Left inferior parietal	-3.97	1.23E-04	0.08
Left pars orbitalis	-3.82	2.11E-04	0.07
Right post central gyrus	-3.67	3.58E-04	0.07

Difference in R^2^ is reported for full model that included age, sex, group and groupxage, versus the reduced model that included age, sex, and group.

Our second analysis compared age-related volume in DS to age-related volume in WS (Table 3; Additional file 1: Table S4). The DS group showed a stronger relationship between age and volume for the left and right lateral ventricles relative to WS, and this difference remained significant after correcting for multiple comparisons. Plots of age versus volume for all significant ROIs are presented in Figures 2 and 3.

**Table 3:** Brain regions where the relationship between age and volume was significantly different between participants with Down syndrome (DS) versus Williams syndrome (WS)

**Region of interest**	**Group (DS versus WS) × Age Interaction Term**		
	***t***	***P*-value**	**Difference in R**^**2**^
Right lateral ventricle	4.42	8.64E-05	0.14
Left lateral ventricle	3.75	1.35E-04	0.12

The right lateral ventricle association was significant after Bonferroni correction.

To investigate whether outliers were driving the observed effects, we removed the participants whose ICV-corrected total gray matter volume was outside of two standard deviations of the grand mean, and we repeated our analyses. One DS subject and four WS adults were removed, but no outliers were identified for the TD group. For the first regression analysis, the interaction model term Group (DS versus TD)x Age, comparing the relationship between volume and age between DS and TD groups, remained significant after Bonferroni correction for the left superior parietal lobe, and in all other ROIs found significant in primary analyses, the interaction term was nominally significant at an unadjusted *P* < .05. For the second regression analysis, the interaction model term comparing age-related effects in volume between the DS and WS groups remained significant after Bonferroni correction for the right lateral ventricle, and for the left lateral ventricle, the interaction term was nominally significant at an unadjusted *P* < .05. No additional ROIs reached Bonferroni significance after outliers were removed in either analysis. These results are presented in Additional file 1: Tables S5a and S5b and Additional file 1: Figure S1.

In secondary analyses, we explored the relationships between brain volume in the eight significant ROIs reported above and the DLD-SOS score, the DLD-SCS score, and *APOE* status in DS adults using linear regression. Full results are presented in Table 4. DLD-SCS and -SOS scores were significantly intercorrelated (r^2^ = .770, *P* = .002) and explained similarly high rates of variability in regional brain volume, beyond that explained by the reduced model including age and sex. DLS-SCS scores explained 21 and 25% of the variance in LILV and RILV volume, respectively (*P* = .042 and *P* = .031; Additional file 1: Figure S2). DLD-SOS scores explained 38% of variance of both the LILV and RILV volumes (*P* = .001 and *P* = .003; Additional file 1: Figure S3). DLD-SOS was also nominally associated with the overall right ventricular volume (*P* = .029) and explained 15% of right ventricular volume variance. *APOE* explained 22% of the variance in LILV and RILV volume (*P* = .026 and .033; Additional file 1: Figure S4).

**Table 4:** **Relationship between Volume and Dementia Questionnaire for People with Learning Disabilities (DLD) sum of cognitive scores (SCS) and sum of social scores (SOS) or *APOE *** **in brain regions showing a correlation with age in Down syndrome participants**

**Region of interest**	**DLD-SCS**			**DLD-SOS**			***APOE***		
	***t***	***P*-value**	**Difference in R**^**2**^	***t***	***P*-value**	**Difference in R**^**2**^	***t***	***P*-value**	**Difference in R**^**2**^
LILV	2.37	*0.042*	0.21	4.72	*0.001*	0.38	2.61	*0.026*	0.22
RILV	2.56	*0.031*	0.25	4.01	*0.003*	0.38	2.47	*0.033*	0.22
Left superior parietal	-1.32	0.219	0.06	0.41	0.693	0.01	-1.06	0.315	0.04
Left pars orbitalis	-1.66	0.131	0.08	-1.32	0.220	0.06	-1.23	0.247	0.10
Left inferior parietal	-1.21	0.256	0.07	-1.59	0.146	0.10	-1.87	0.091	0.06
Right post central gyrus	-0.54	0.604	0.01	-1.11	0.294	0.05	0.05	0.961	0.00
Left ventricle	0.57	0.582	0.01	2.06	0.069	0.13	1.45	0.178	0.07
Right ventricle	0.91	0.386	0.03	2.59	*0.029*	0.15	1.78	0.105	0.08

Italicized *P*-values indicate nominal association at uncorrected *P* < .05. The Difference in R^2^ values indicates the difference in the variability in the region of interest (ROI) volume explained by the full model that included the predictor of interest (DLD-SCS, DLD-SOS, or APOE), compared to that explained by the reduced model with only age and sex as predictors.

Thus, in summary, all three variables were nominally associated with LILV and RILV volume (*P* < .05), and the association between DLD-SOS and RILV volume was significant after Bonferroni correction. However, after the one DS outlier was removed, only the relationships between DLD-SCS and RILV and between DLD-SOS and LILV remained even nominally significant (*P* = .037 and .009, respectively). Notably, the removed subject was one of three adults who had an *APOE* ϵ4 allele.

## Discussion

The first aim of the current study was to confirm previous findings of age-related brain changes in DS [1]. In previous studies, adults with DS had significantly stronger relationships between age and volume in the frontal, parietal, and temporal lobes, and the lateral ventricles [1]. We replicated these findings in the frontal lobe (specifically, the left pars orbitalis gyri frontalis inferioris), the parietal lobe (specifically, the left superior and left inferior parietal cortices and the right post central gyrus), and in the lateral ventricles (specifically, the LILV and RILV).

Interestingly, atrophy in these gray matter regions and dilation of the ventricles may be explained by the link between DS and Alzheimer’s disease (AD). Individuals with DS are at a greatly increased risk of developing AD, with up to 70 percent developing dementia by the age of 70 [36]. In fact, adults with DS account for up to 60% of individuals with developmental disabilities who exhibit signs of AD [37]. The risk for AD in DS is primarily related to triplication of the amyloid precursor protein (*APP*), which is on chromosome 21 [38]. However, one’s genotype at the apolipoprotein E (*APOE*) gene, whose protein product is involved in the processing of amyloid beta isoforms, has also been shown to modulate risk for developing AD in the DS population [39,40]. Postmortem studies have revealed that plaque load in adults with DS increases with age: by 40 years of age, nearly all individuals with DS have amyloid beta plaques in the brain [41], and this characteristic feature of AD has neurotoxic effects that can lead to neurodegeneration and loss in brain volume [42]. More specifically, both the frontal and parietal lobes have shown increased amyloid load as measured by positron emission tomography (PET) in participants with DS [43]. Therefore, the age-related effects detected in this study may be due to the early-onset of neurotoxic effects related to increases in amyloid load in adults with DS.

Age-related volume in DS relative to WS adults was significant in the left and right total lateral ventricles, even after Bonferroni correction for multiple comparisons, and there was a stronger relationship between age and volume in the inferior lateral ventricles in the DS group compared to the TD group. These results are particularly interesting given the high prevalence of AD in DS and the association between ventricular dilation and AD. Volume of the lateral ventricles has repeatedly shown a relationship to AD status and disease progression in the general population [44-47]. The lateral ventricles normally dilate over time with age, as brain tissue volume decreases, but in patients with mild cognitive impairment (MCI) or AD, the rate of ventricular dilation is much greater than in the general aging population [48]. The inferior lateral ventricles are surrounded by subcortical gray matter structures, and these structures, particularly the hippocampus, entorhinal cortex, and amygdala, accumulate amyloid plaques and exhibit atrophy in AD [48,49] and DS [50-54]. Since ventricular dilation is cumulatively and inversely reflective of atrophy of these surrounding structures [55], and since we found this strong relationship between age and ventricular volume was not present in WS, these results may be reflective of the neurodegenerative effects of AD pathology on the structures surrounding the ventricles.

We also investigated whether, similar to the DS group, the WS group experienced greater age-related effects compared to the TD group, but we did not find evidence to support this hypothesis. This is in line with the one previously reported finding of aging WS adults [20]. However, this may be due to the difference in age ranges between the WS and TD groups (Table 1), and further investigation of age-related volume in WS in a larger study, ideally with longitudinal data, is warranted.

As a secondary exploratory analysis in the DS cohort only, regions with significant age-related changes (in DS versus TD and DS versus WS) were evaluated for association with DLD-SOS, DLD-SCS and *APOE* ϵ4 status. We observed a strong relationship between ILV volume and both the cognitive and social scores on the DLD, a test that measures dementia-related impairments in the DS population, though only the relationship between RILV and DLD-SCS and between LILV and DLD-SOS remained nominally significant after the one DS outlier was removed. While we have collected data from DS study participants using a comprehensive battery of tests, we do not have the clinical expertise and have not sought consensus from clinical experts to determine clinical dementia status. Instead, we have used the DLD scores as a quantitative proxy measure of behavioral symptoms related to dementia status. Previous studies have shown that individuals with DS have decreased regional brain volumes with onset of dementia [50,52,53,56], and in the current study, we observed that higher levels of dementia symptomology (as measured by the DLD) were associated with greater ventricular volume, an MRI biomarker of neurodegeneration.

*APOE* is a very strong genetic risk factor predisposing patients to AD-associated neurodegeneration; TD adults who are carriers of the ϵ4 risk allele show more signs of neurodegeneration before symptom onset [57]. While *APOE* is also known to be associated with further increased risk of AD in the DS population, to our knowledge, this is the first study in DS to investigate the association of *APOE* ϵ4 carrier status with MRI volume data [39]. The observed ϵ4 carrier frequency was 3/28 alleles, or 11%, which is similar to the frequency observed in the general population (13%). However, one of the three ϵ4 carriers was the DS group outlier, and it was this subject whose data drove the observed effect on brain volume by *APOE* genotype. Thus, we are not able to make a strong conclusion based on these data, and future analyses with larger sample sizes will be necessary to confirm an effect of *APOE* on age-related differences in brain volume in DS.

The present results must be interpreted within the framework of our statistical models. The WS and DS groups differed in mean age, but in all cases, we included age and sex as important covariates known to be related to neurodegeneration. The highly significant *P*-values we observed seem to be driven in part by one DS outlier, but the trends did not change when the outliers were removed (Additional file 1: Figure S1). Furthermore, though this study found a significant age-related difference in DS adults in AD-related regions, we may have been underpowered to detect more subtle AD-related changes due to 1) the relatively small sample size of the DS group, 2) the strict statistical threshold used for significance, and 3) the fact that FreeSurfer parameter estimates for smaller subcortical areas, such as the entorhinal cortex and hippocampus, are known to exhibit greater error [58]. Despite this, some AD-related regions were nominally significant at an unadjusted *P* < .05 (left hippocampus: *P* = .0023, right amygdala: *P* = .0042, left amygdala: *P* = .0042, right posterior cingulate gyrus: *P* = .0279, left posterior cingulate gyrus: *P* = .0495). These results warrant further analysis of age-related affects in AD-related regions in a larger cohort of DS adults.

## Conclusion

In conclusion, individuals with DS demonstrated a unique pattern of age-related effects on gray matter and ventricular volume, the latter of which was associated with dementia rating scores in the DS group. Results may indicate that early onset of brain aging in DS is primarily due to DS-specific neurodegenerative processes, as opposed to general atypical neurodevelopment.

## Abbreviations

AD: Alzheimer’s disease; APOE: apolipoprotein-E gene; DLD: Dementia Questionnaire for People with Learning Disabilities; DLD-SOS: Sum of Social Scores for DLD; DLD-SCS: Sum of Cognitive Scores for DLD; DS: Down syndrome; ICV: (total) intracranial volume; LILV: left inferior lateral ventricle; MCI: mild cognitive impairment; MRI: magnetic resonance imaging; PET: positron emission tomography; RILV: right inferior lateral ventricle; ROI: (brain) region of interest; SNP: single nucleotide polymorphism; TD: typical development; WS: Williams syndrome.

## Competing interests

The authors declare that they have no competing interests.

## Authors’ contributions

MEK contributed to study design, carried out the statistical analyses, participated in subject recruitment, scanning, imaging analysis and quality control, and drafted the manuscript. CME contributed to imaging analysis and quality control and helped to draft the manuscript. TJH contributed to study design, participated in its coordination, and helped to draft the manuscript. JNV and JP participated in subject recruitment, scanning, and imaging analysis. LES, GC, and LVR contributed to participant recruitment, neuropsychological testing, and genotyping. SAM participated in scanning and imaging analysis. ND contributed to imaging analysis and quality control. SNA and JUB participated in subject recruitment and scanning and helped to draft the manuscript. EMD participated in the study design and helped to draft the manuscript. TATW conceived of the study, participated in its design and the coordination of subject recruitment, scanning and imaging quality control, and helped to draft the manuscript. All authors read and approved the final manuscript.

## Supplementary Material

Additional file 1: Table S1a Regions of interest included in analysis. **Table S1b.** Regions of interest not included in analysis. **Table S2.** Additional demographics for Down syndrome participants, including *APOE* ϵ4 genotype, test scores for the Dementia Questionnaire for People with Learning Disabilities (DLD) (sum of cognitive scores (SCS) and sum of social scores (SOS)), and the Kaufman Brief Intelligence Test (KBIT) composite score. **Table S3.** complete results from analysis of relationship between volume and age comparing the Down syndrome (DS) and Williams syndrome (WS) groups to the typically developing (TD) controls. **Table S4.** Results from analysis comparing relationship between volume and age between participants with Down syndrome and Williams syndrome. **Table S5.** To investigate whether the observed effects were being driven by outliers, we removed the subjects whose ICV-corrected total gray matter volume was outside of two standard deviations of the grand mean and re-ran our analyses. **Figure S1.** This figure shows results from *post hoc* analyses which excluded subjects whose ICV-normalized gray matter volumes fell outside two standard deviations of the grand mean for their respective group (one with DS and four with WS). **Figure S2.** regional brain volumes normalized to ICV are plotted in relationship to Dementia Questionnaire for People with Learning Disabilities (DLD) sum of cognitive scores (SCS) across the Down syndrome subject group in: **(a)** right inferior lateral ventricle (RILV) and **(b)** left inferior lateral ventricle (LILV). **Figure S3.** Regional brain volumes normalized to ICV are plotted in relationship to Dementia Questionnaire for People with Learning Disabilities (DLD) sum of social scores (SOS) across the Down syndrome subject group in: **(a)** right inferior lateral ventricle (RILV) and **(b)** left inferior lateral ventricle (LILV). **Figure S4.** While *APOE* ϵ4 carrier status showed a trend for association with regional brain volume, a larger sample size will be needed to accurately estimate this effect. Click here for file
