# Supplementary material for: Differences in age-related effects on brain volume in Down syndrome as compared to Williams syndrome and typical development
Source: J Neurodev Disord. 2014 Apr 9;6(1):8. doi: 10.1186/1866-1955-6-8 (PMC4022321; doi:10.1186/1866-1955-6-8)

# Supplementary Materials

## Supplementary Table 1a: Regions of interest included in analysis

## Supplementary Table 1b Regions of interest not included in analysis

## Supplementary Table 2: Additional demographics for Down syndrome participants, including *APOE* ε4 genotype, test scores for the Dementia Questionnaire for People with Learning Disabilities (DLD) (sum of cognitive scores (SCS) and sum of social scores (SOS)), and the Kaufman Brief Intelligence Test (KBIT) composite score.

| **Age** | **Sex** | **APOE** | **DLD-SCS** | **DLD-SOS** | **KBIT Composite** |
| --- | --- | --- | --- | --- | --- |
| 19 | Female | e2/e3 | 0 | 1 | 49 |
| 20 | Male | e2/e3 | 0 | 3 | 50 |
| 23 | Male | e3/e3 | 0 | 4 | 50 |
| 33 | Female | e3/e4 | 0 | 10 | 40 |
| 34 | Male | e3/e3 | 8 | 5 | 42 |
| 35 | Female | e3/e3 | 0 | 8 | 47 |
| 37 | Female | e3/e3 | 0 | 2 | 57 |
| 39 | Female | e3/e3 | 0 | 1 | 61 |
| 41 | Female | e3/e4 | 6 | 5 | 54 |
| 43 | Female | e3/e3 | 0 | 0 | 40 |
| 49 | Male | e3/e3 | 1 | 9 | 40 |
| 54 | Male | e3/e4 | 21 | 18 | 40 |
| 56 | Male | e3/e3 | 20 | 10 | 40 |
| 63 | Male | e3/e3 | 12 | 5 | 40 |

## Supplementary Table 3

Complete results from analysis of relationship between volume and age comparing the Down syndrome (DS) and Williams syndrome (WS) groups to the typically developing (TD) controls. Results are sorted first by contrast group (DS, then WS) and then in ascending order of p-value.

|  | **Group vs TD x Age Interaction** | | |
| --- | --- | --- | --- |
| ROI | Group | t | p-value |
| LeftInfLatVent | DS | 4.32 | 3.21E-05 |
| RightInfLatVent | DS | 4.05 | 8.85E-05 |
| lh_superiorparietal_volume | DS | -4.04 | 9.26E-05 |
| lh_inferiorparietal_volume | DS | -3.98 | 1.16E-04 |
| lh_parsorbitalis_volume | DS | -3.81 | 2.21E-04 |
| rh_postcentral_volume | DS | -3.67 | 3.56E-04 |
| lh_precuneus_volume | DS | -3.39 | 9.29E-04 |
| lh_medialorbitofrontal_volume | DS | -3.37 | 1.01E-03 |
| LeftPallidum | DS | -3.28 | 1.35E-03 |
| rh_cuneus_volume | DS | -3.25 | 1.46E-03 |
| rh_superiorfrontal_volume | DS | -3.25 | 1.46E-03 |
| RightPallidum | DS | -3.22 | 1.61E-03 |
| lh_isthmuscingulate_volume | DS | -3.18 | 1.84E-03 |
| rh_lateralorbitofrontal_volume | DS | -3.14 | 2.09E-03 |
| lh_parstriangularis_volume | DS | -3.13 | 2.21E-03 |
| LeftHippocampus | DS | -3.11 | 2.30E-03 |
| RightLateralVentricle | DS | 3.05 | 2.78E-03 |
| rh_paracentral_volume | DS | -3.02 | 3.10E-03 |
| rh_parsorbitalis_volume | DS | -3.00 | 3.28E-03 |
| RightThalamusProper | DS | -3.00 | 3.31E-03 |
| CortexVol | DS | -2.97 | 3.60E-03 |
| CC_Mid_Anterior | DS | -2.95 | 3.80E-03 |
| rh_rostralmiddlefrontal_volume | DS | -2.95 | 3.82E-03 |
| lhCortexVol | DS | -2.94 | 3.89E-03 |
| RightAmygdala | DS | -2.91 | 4.22E-03 |
| LeftAmygdala | DS | -2.91 | 4.23E-03 |
| rh_precuneus_volume | DS | -2.91 | 4.33E-03 |
| lh_postcentral_volume | DS | -2.86 | 4.97E-03 |
| lh_rostralmiddlefrontal_volume | DS | -2.85 | 5.09E-03 |
| LeftLateralVentricle | DS | 2.78 | 6.27E-03 |
| rh_entorhinal_volume | DS | -2.78 | 6.37E-03 |
| RightAccumbensarea | DS | -2.77 | 6.46E-03 |
| lh_middletemporal_volume | DS | -2.76 | 6.58E-03 |
| rh_temporalpole_volume | DS | -2.73 | 7.20E-03 |
| rh_medialorbitofrontal_volume | DS | -2.73 | 7.20E-03 |
| lh_paracentral_volume | DS | -2.71 | 7.60E-03 |
| lh_lateralorbitofrontal_volume | DS | -2.69 | 8.08E-03 |
| LeftThalamusProper | DS | -2.69 | 8.22E-03 |
| rh_frontalpole_volume | DS | -2.69 | 8.23E-03 |
| rh_parstriangularis_volume | DS | -2.62 | 9.97E-03 |
| rh_isthmuscingulate_volume | DS | -2.61 | 1.00E-02 |
| rh_inferiorparietal_volume | DS | -2.58 | 1.11E-02 |
| lh_parahippocampal_volume | DS | -2.57 | 1.13E-02 |
| rh_superiortemporal_volume | DS | -2.53 | 1.27E-02 |
| rh_insula_volume | DS | -2.47 | 1.50E-02 |
| lh_superiorfrontal_volume | DS | -2.45 | 1.58E-02 |
| lh_fusiform_volume | DS | -2.43 | 1.66E-02 |
| lh_parsopercularis_volume | DS | -2.33 | 2.12E-02 |
| lh_lateraloccipital_volume | DS | -2.32 | 2.22E-02 |
| rh_lateraloccipital_volume | DS | -2.25 | 2.60E-02 |
| rh_posteriorcingulate_volume | DS | -2.22 | 2.79E-02 |
| rh_fusiform_volume | DS | -2.17 | 3.21E-02 |
| lh_superiortemporal_volume | DS | -2.13 | 3.49E-02 |
| rh_middletemporal_volume | DS | -2.02 | 4.52E-02 |
| lh_posteriorcingulate_volume | DS | -1.98 | 4.95E-02 |
| LeftPutamen | DS | -1.95 | 5.36E-02 |
| LeftCaudate | DS | -1.94 | 5.51E-02 |
| CorticalWhiteMatterVol | DS | -1.93 | 5.54E-02 |
| rh_pericalcarine_volume | DS | -1.92 | 5.67E-02 |
| rh_superiorparietal_volume | DS | -1.92 | 5.68E-02 |
| CC_Anterior | DS | -1.91 | 5.84E-02 |
| rh_inferiortemporal_volume | DS | -1.90 | 5.95E-02 |
| lh_inferiortemporal_volume | DS | -1.89 | 6.16E-02 |
| rh_supramarginal_volume | DS | -1.84 | 6.76E-02 |
| RightPutamen | DS | -1.82 | 7.07E-02 |
| rh_bankssts_volume | DS | -1.81 | 7.22E-02 |
| lh_insula_volume | DS | -1.81 | 7.24E-02 |
| rh_caudalanteriorcingulate_volume | DS | -1.74 | 8.48E-02 |
| rh_parsopercularis_volume | DS | -1.70 | 9.22E-02 |
| RightCaudate | DS | -1.69 | 9.26E-02 |
| LeftVentralDC | DS | -1.68 | 9.64E-02 |
| rh_lingual_volume | DS | -1.67 | 9.65E-02 |
| lh_cuneus_volume | DS | -1.67 | 9.69E-02 |
| rh_parahippocampal_volume | DS | -1.67 | 9.80E-02 |
| rh_transversetemporal_volume | DS | -1.66 | 9.86E-02 |
| lh_rostralanteriorcingulate_volume | DS | -1.66 | 9.97E-02 |
| LeftAccumbensarea | DS | -1.64 | 1.03E-01 |
| RightVentralDC | DS | -1.63 | 1.06E-01 |
| lh_caudalanteriorcingulate_volume | DS | -1.59 | 1.14E-01 |
| lh_temporalpole_volume | DS | -1.56 | 1.21E-01 |
| CC_Central | DS | -1.55 | 1.23E-01 |
| lh_lingual_volume | DS | -1.51 | 1.34E-01 |
| BrainStem | DS | -1.49 | 1.39E-01 |
| rh_precentral_volume | DS | -1.46 | 1.46E-01 |
| RightHippocampus | DS | -1.42 | 1.59E-01 |
| lh_transversetemporal_volume | DS | -1.38 | 1.69E-01 |
| CC_Posterior | DS | -1.31 | 1.94E-01 |
| lh_pericalcarine_volume | DS | -1.27 | 2.06E-01 |
| CC_Mid_Posterior | DS | -1.22 | 2.24E-01 |
| RightCerebellumCortex | DS | -1.13 | 2.59E-01 |
| lh_precentral_volume | DS | -1.11 | 2.71E-01 |
| lh_supramarginal_volume | DS | -1.10 | 2.73E-01 |
| lh_bankssts_volume | DS | -1.08 | 2.84E-01 |
| rh_rostralanteriorcingulate_volume | DS | -1.06 | 2.93E-01 |
| LeftCerebellumCortex | DS | -0.93 | 3.54E-01 |
| lh_frontalpole_volume | DS | -0.93 | 3.57E-01 |
| lh_entorhinal_volume | DS | -0.91 | 3.62E-01 |
| lh_caudalmiddlefrontal_volume | DS | -0.87 | 3.84E-01 |
| LeftCerebellumWhiteMatter | DS | -0.83 | 4.08E-01 |
| rh_caudalmiddlefrontal_volume | DS | -0.80 | 4.24E-01 |
| RightCerebellumWhiteMatter | DS | -0.71 | 4.78E-01 |
| rh_medialorbitofrontal_volume | WS | -2.37 | 1.94E-02 |
| rh_entorhinal_volume | WS | -1.93 | 5.63E-02 |
| lh_superiorparietal_volume | WS | -1.91 | 5.84E-02 |
| lh_parstriangularis_volume | WS | -1.85 | 6.72E-02 |
| rh_superiorfrontal_volume | WS | -1.60 | 1.11E-01 |
| RightVentralDC | WS | -1.55 | 1.23E-01 |
| rh_temporalpole_volume | WS | -1.54 | 1.26E-01 |
| rh_parstriangularis_volume | WS | -1.53 | 1.29E-01 |
| rh_bankssts_volume | WS | -1.51 | 1.34E-01 |
| rh_superiorparietal_volume | WS | -1.48 | 1.41E-01 |
| lh_paracentral_volume | WS | -1.47 | 1.44E-01 |
| rh_parsorbitalis_volume | WS | -1.44 | 1.53E-01 |
| lh_entorhinal_volume | WS | -1.42 | 1.57E-01 |
| LeftCerebellumWhiteMatter | WS | 1.39 | 1.67E-01 |
| rh_fusiform_volume | WS | -1.36 | 1.77E-01 |
| RightCerebellumWhiteMatter | WS | 1.34 | 1.81E-01 |
| LeftCerebellumCortex | WS | -1.30 | 1.95E-01 |
| lh_parsorbitalis_volume | WS | -1.30 | 1.97E-01 |
| lh_precuneus_volume | WS | -1.28 | 2.02E-01 |
| lh_caudalanteriorcingulate_volume | WS | -1.27 | 2.06E-01 |
| RightCerebellumCortex | WS | -1.24 | 2.19E-01 |
| rh_paracentral_volume | WS | -1.21 | 2.28E-01 |
| CortexVol | WS | -1.20 | 2.33E-01 |
| lh_fusiform_volume | WS | -1.19 | 2.35E-01 |
| lh_rostralanteriorcingulate_volume | WS | -1.19 | 2.35E-01 |
| lh_precentral_volume | WS | -1.19 | 2.36E-01 |
| lh_superiorfrontal_volume | WS | -1.17 | 2.46E-01 |
| RightInfLatVent | WS | -1.16 | 2.47E-01 |
| lhCortexVol | WS | -1.16 | 2.48E-01 |
| lh_inferiorparietal_volume | WS | -1.09 | 2.77E-01 |
| lh_lateralorbitofrontal_volume | WS | -1.09 | 2.78E-01 |
| lh_cuneus_volume | WS | -1.08 | 2.81E-01 |
| rh_postcentral_volume | WS | -1.07 | 2.85E-01 |
| rh_inferiortemporal_volume | WS | -1.07 | 2.87E-01 |
| CC_Mid_Anterior | WS | 1.06 | 2.90E-01 |
| rh_inferiorparietal_volume | WS | -1.04 | 2.99E-01 |
| lh_supramarginal_volume | WS | -1.04 | 2.99E-01 |
| lh_parsopercularis_volume | WS | -1.03 | 3.03E-01 |
| rh_precuneus_volume | WS | -1.01 | 3.13E-01 |
| rh_rostralmiddlefrontal_volume | WS | -1.01 | 3.14E-01 |
| LeftVentralDC | WS | -1.01 | 3.15E-01 |
| RightAccumbensarea | WS | -0.99 | 3.22E-01 |
| rh_precentral_volume | WS | -0.96 | 3.40E-01 |
| rh_frontalpole_volume | WS | -0.94 | 3.50E-01 |
| lh_insula_volume | WS | -0.92 | 3.59E-01 |
| rh_supramarginal_volume | WS | -0.91 | 3.64E-01 |
| RightLateralVentricle | WS | -0.90 | 3.70E-01 |
| rh_middletemporal_volume | WS | -0.90 | 3.71E-01 |
| lh_middletemporal_volume | WS | -0.85 | 3.94E-01 |
| LeftAccumbensarea | WS | -0.85 | 3.97E-01 |
| rh_isthmuscingulate_volume | WS | -0.84 | 4.00E-01 |
| rh_parahippocampal_volume | WS | -0.84 | 4.01E-01 |
| lh_rostralmiddlefrontal_volume | WS | -0.82 | 4.14E-01 |
| LeftLateralVentricle | WS | -0.79 | 4.33E-01 |
| lh_superiortemporal_volume | WS | -0.75 | 4.57E-01 |
| rh_lateraloccipital_volume | WS | -0.74 | 4.62E-01 |
| lh_posteriorcingulate_volume | WS | -0.69 | 4.91E-01 |
| rh_lateralorbitofrontal_volume | WS | -0.68 | 4.97E-01 |
| LeftCaudate | WS | -0.68 | 4.98E-01 |
| CC_Central | WS | 0.66 | 5.13E-01 |
| RightThalamusProper | WS | -0.65 | 5.16E-01 |
| RightPallidum | WS | 0.64 | 5.23E-01 |
| rh_cuneus_volume | WS | -0.63 | 5.27E-01 |
| lh_parahippocampal_volume | WS | -0.62 | 5.33E-01 |
| lh_lateraloccipital_volume | WS | -0.62 | 5.38E-01 |
| lh_lingual_volume | WS | -0.62 | 5.38E-01 |
| LeftPutamen | WS | -0.60 | 5.47E-01 |
| rh_superiortemporal_volume | WS | -0.59 | 5.59E-01 |
| RightPutamen | WS | -0.58 | 5.64E-01 |
| lh_medialorbitofrontal_volume | WS | -0.58 | 5.64E-01 |
| lh_caudalmiddlefrontal_volume | WS | -0.57 | 5.71E-01 |
| RightCaudate | WS | -0.53 | 5.96E-01 |
| rh_insula_volume | WS | -0.50 | 6.15E-01 |
| rh_lingual_volume | WS | -0.47 | 6.37E-01 |
| rh_transversetemporal_volume | WS | 0.47 | 6.39E-01 |
| rh_caudalanteriorcingulate_volume | WS | -0.44 | 6.62E-01 |
| RightAmygdala | WS | 0.44 | 6.62E-01 |
| rh_posteriorcingulate_volume | WS | -0.41 | 6.82E-01 |
| lh_temporalpole_volume | WS | -0.41 | 6.83E-01 |
| CorticalWhiteMatterVol | WS | -0.31 | 7.60E-01 |
| CC_Posterior | WS | -0.29 | 7.76E-01 |
| LeftPallidum | WS | -0.27 | 7.84E-01 |
| BrainStem | WS | -0.26 | 7.93E-01 |
| LeftInfLatVent | WS | -0.26 | 7.96E-01 |
| lh_frontalpole_volume | WS | -0.26 | 7.97E-01 |
| lh_bankssts_volume | WS | -0.25 | 8.04E-01 |
| lh_isthmuscingulate_volume | WS | -0.24 | 8.12E-01 |
| RightHippocampus | WS | -0.24 | 8.14E-01 |
| CC_Mid_Posterior | WS | 0.20 | 8.46E-01 |
| lh_pericalcarine_volume | WS | -0.19 | 8.48E-01 |
| lh_postcentral_volume | WS | -0.19 | 8.48E-01 |
| LeftThalamusProper | WS | 0.16 | 8.70E-01 |
| lh_transversetemporal_volume | WS | 0.15 | 8.79E-01 |
| rh_pericalcarine_volume | WS | -0.14 | 8.85E-01 |
| LeftHippocampus | WS | 0.13 | 8.95E-01 |
| rh_caudalmiddlefrontal_volume | WS | 0.12 | 9.03E-01 |
| rh_rostralanteriorcingulate_volume | WS | -0.07 | 9.45E-01 |
| CC_Anterior | WS | 0.05 | 9.58E-01 |
| lh_inferiortemporal_volume | WS | -0.03 | 9.76E-01 |
| LeftAmygdala | WS | 0.03 | 9.77E-01 |
| rh_parsopercularis_volume | WS | -0.01 | 9.92E-01 |

## Supplementary Table 4

Results from analysis comparing relationship between volume and age between participants with Down syndrome and Williams syndrome.

|  | **Group (DS v WS) x Age Interaction** | |
| --- | --- | --- |
| **ROI** | **t** | **p-value** |
| RightLateralVentricle | 4.30 | 8.64E-05 |
| LeftLateralVentricle | 4.16 | 1.35E-04 |
| RightInfLatVent | 2.93 | 5.25E-03 |
| RightPallidum | -2.63 | 1.14E-02 |
| LeftInfLatVent | 2.55 | 1.42E-02 |
| lh_parstriangularis_volume | -2.51 | 1.57E-02 |
| LeftThalamusProper | -2.30 | 2.61E-02 |
| rh_parstriangularis_volume | -2.13 | 3.83E-02 |
| CC_Mid_Anterior | -2.11 | 4.05E-02 |
| LeftPallidum | -2.10 | 4.15E-02 |
| rh_bankssts_volume | -2.07 | 4.36E-02 |
| rh_medialorbitofrontal_volume | -2.07 | 4.38E-02 |
| lh_isthmuscingulate_volume | -2.06 | 4.52E-02 |
| LeftHippocampus | -2.04 | 4.67E-02 |
| RightThalamusProper | -2.04 | 4.71E-02 |
| 4thVentricle | 2.01 | 5.05E-02 |
| RightAmygdala | -1.96 | 5.57E-02 |
| lh_inferiorparietal_volume | -1.85 | 7.08E-02 |
| lh_superiorparietal_volume | -1.78 | 8.14E-02 |
| rh_superiorfrontal_volume | -1.77 | 8.39E-02 |
| lh_medialorbitofrontal_volume | -1.76 | 8.57E-02 |
| lh_postcentral_volume | -1.75 | 8.65E-02 |
| rh_postcentral_volume | -1.72 | 9.17E-02 |
| LeftAmygdala | -1.70 | 9.49E-02 |
| rh_cuneus_volume | -1.69 | 9.84E-02 |
| lh_superiorparietal_volume | -1.63 | 1.09E-01 |
| lh_parsorbitalis_volume | -1.63 | 1.10E-01 |
| lh_fusiform_volume | -1.57 | 1.23E-01 |
| 3rdVentricle | 1.56 | 1.25E-01 |
| lh_precuneus_volume | -1.56 | 1.26E-01 |
| lh_precuneus_volume | -1.54 | 1.29E-01 |
| rh_lateralorbitofrontal_volume | -1.53 | 1.33E-01 |
| lh_supramarginal_volume | -1.53 | 1.33E-01 |
| LeftCerebellumWhiteMatter | 1.53 | 1.33E-01 |
| rh_rostralmiddlefrontal_volume | -1.51 | 1.38E-01 |
| rh_lingual_volume | -1.50 | 1.40E-01 |
| lh_rostralmiddlefrontal_volume | -1.49 | 1.43E-01 |
| RightAccumbensarea | -1.48 | 1.45E-01 |
| LeftPutamen | -1.46 | 1.50E-01 |
| rh_fusiform_volume | -1.45 | 1.53E-01 |
| rh_inferiorparietal_volume | -1.45 | 1.55E-01 |
| LeftCerebellumCortex | -1.44 | 1.55E-01 |
| rh_insula_volume | -1.44 | 1.56E-01 |
| lh_superiorfrontal_volume | -1.44 | 1.57E-01 |
| rh_rostralmiddlefrontal_volume | -1.43 | 1.61E-01 |
| CC_Central | -1.42 | 1.61E-01 |
| rh_precuneus_volume | -1.42 | 1.62E-01 |
| LeftCerebellumWhiteMatter | -1.42 | 1.63E-01 |
| rh_parsorbitalis_volume | -1.41 | 1.67E-01 |
| 3rdVentricle | 1.39 | 1.71E-01 |
| CortexVol | -1.38 | 1.75E-01 |
| CC_Anterior | -1.36 | 1.81E-01 |
| rh_transversetemporal_volume | -1.35 | 1.83E-01 |
| rh_superiorparietal_volume | -1.34 | 1.87E-01 |
| RightPutamen | -1.32 | 1.95E-01 |
| rh_superiortemporal_volume | -1.31 | 1.96E-01 |
| rh_middletemporal_volume | -1.31 | 1.97E-01 |
| lh_parsorbitalis_volume | -1.30 | 2.01E-01 |
| RightCerebellumWhiteMatter | 1.30 | 2.01E-01 |
| RightVentralDC | -1.29 | 2.03E-01 |
| lh_middletemporal_volume | -1.29 | 2.04E-01 |
| RightAccumbensarea | -1.28 | 2.05E-01 |
| rh_pericalcarine_volume | -1.28 | 2.07E-01 |
| lh_lateralorbitofrontal_volume | -1.28 | 2.08E-01 |
| lh_entorhinal_volume | -1.27 | 2.09E-01 |
| rh_inferiortemporal_volume | -1.26 | 2.13E-01 |
| rh_isthmuscingulate_volume | -1.26 | 2.14E-01 |
| rh_parsopercularis_volume | -1.25 | 2.16E-01 |
| lh_rostralanteriorcingulate_volume | -1.25 | 2.17E-01 |
| CorticalWhiteMatterVol | -1.25 | 2.18E-01 |
| rh_precuneus_volume | -1.24 | 2.21E-01 |
| CortexVol | -1.24 | 2.21E-01 |
| lh_caudalanteriorcingulate_volume | -1.22 | 2.29E-01 |
| lh_paracentral_volume | -1.22 | 2.29E-01 |
| lh_parahippocampal_volume | -1.22 | 2.29E-01 |
| lh_rostralmiddlefrontal_volume | -1.21 | 2.31E-01 |
| RightCerebellumWhiteMatter | -1.20 | 2.35E-01 |
| rh_paracentral_volume | -1.19 | 2.40E-01 |
| LeftAccumbensarea | -1.18 | 2.42E-01 |
| rh_posteriorcingulate_volume | -1.18 | 2.44E-01 |
| rh_paracentral_volume | -1.17 | 2.47E-01 |
| BrainStem | -1.17 | 2.47E-01 |
| rh_frontalpole_volume | -1.17 | 2.49E-01 |
| rh_supramarginal_volume | -1.17 | 2.49E-01 |
| rh_entorhinal_volume | -1.16 | 2.54E-01 |
| lh_inferiorparietal_volume | -1.15 | 2.54E-01 |
| RightCerebellumCortex | -1.15 | 2.54E-01 |
| lh_precentral_volume | -1.15 | 2.58E-01 |
| lh_inferiortemporal_volume | -1.14 | 2.59E-01 |
| lh_posteriorcingulate_volume | -1.14 | 2.62E-01 |
| lh_lateraloccipital_volume | -1.13 | 2.63E-01 |
| rh_temporalpole_volume | -1.13 | 2.65E-01 |
| lh_cuneus_volume | -1.12 | 2.67E-01 |
| rh_superiorfrontal_volume | -1.12 | 2.70E-01 |
| lh_middletemporal_volume | -1.10 | 2.76E-01 |
| lh_insula_volume | -1.08 | 2.85E-01 |
| rh_postcentral_volume | -1.08 | 2.87E-01 |
| lh_parstriangularis_volume | -1.07 | 2.88E-01 |
| lh_parsopercularis_volume | -1.07 | 2.90E-01 |
| lh_lateralorbitofrontal_volume | -1.07 | 2.90E-01 |
| rh_inferiorparietal_volume | -1.07 | 2.91E-01 |
| rh_precentral_volume | -1.06 | 2.95E-01 |
| rh_parsorbitalis_volume | -1.04 | 3.05E-01 |
| rh_parstriangularis_volume | -1.02 | 3.13E-01 |
| lh_transversetemporal_volume | -1.02 | 3.15E-01 |
| rh_parahippocampal_volume | -1.01 | 3.17E-01 |
| lh_lingual_volume | -1.01 | 3.19E-01 |
| rh_superiortemporal_volume | -1.00 | 3.23E-01 |
| LeftCaudate | -0.98 | 3.34E-01 |
| lh_superiortemporal_volume | -0.96 | 3.44E-01 |
| CC_Mid_Posterior | -0.95 | 3.46E-01 |
| rh_lateraloccipital_volume | -0.95 | 3.48E-01 |
| LeftPutamen | -0.95 | 3.49E-01 |
| lh_parsopercularis_volume | -0.94 | 3.52E-01 |
| lh_fusiform_volume | -0.93 | 3.57E-01 |
| lh_superiortemporal_volume | -0.93 | 3.58E-01 |
| lh_posteriorcingulate_volume | -0.92 | 3.62E-01 |
| RightLateralVentricle | 0.91 | 3.67E-01 |
| RightPutamen | -0.90 | 3.73E-01 |
| lh_superiorfrontal_volume | -0.89 | 3.78E-01 |
| rh_caudalanteriorcingulate_volume | -0.89 | 3.79E-01 |
| lh_isthmuscingulate_volume | -0.88 | 3.81E-01 |
| rh_lateralorbitofrontal_volume | -0.87 | 3.87E-01 |
| LeftPallidum | -0.87 | 3.91E-01 |
| RightCaudate | -0.86 | 3.94E-01 |
| lh_pericalcarine_volume | -0.85 | 3.98E-01 |
| 4thVentricle | -0.85 | 3.99E-01 |
| LeftVentralDC | -0.85 | 4.00E-01 |
| LeftLateralVentricle | 0.84 | 4.05E-01 |
| rh_temporalpole_volume | -0.82 | 4.14E-01 |
| lh_temporalpole_volume | -0.81 | 4.23E-01 |
| rh_frontalpole_volume | -0.79 | 4.35E-01 |
| lh_caudalmiddlefrontal_volume | -0.77 | 4.43E-01 |
| rh_middletemporal_volume | -0.77 | 4.45E-01 |
| rh_cuneus_volume | -0.76 | 4.51E-01 |
| lh_paracentral_volume | -0.76 | 4.53E-01 |
| lh_parahippocampal_volume | -0.73 | 4.67E-01 |
| rh_isthmuscingulate_volume | -0.73 | 4.69E-01 |
| RightHippocampus | -0.73 | 4.70E-01 |
| rh_rostralanteriorcingulate_volume | -0.72 | 4.76E-01 |
| rh_supramarginal_volume | -0.71 | 4.80E-01 |
| lh_bankssts_volume | -0.71 | 4.81E-01 |
| rh_lateraloccipital_volume | -0.68 | 5.03E-01 |
| rh_posteriorcingulate_volume | -0.67 | 5.08E-01 |
| CC_Mid_Anterior | 0.65 | 5.16E-01 |
| rh_caudalmiddlefrontal_volume | -0.65 | 5.19E-01 |
| lh_insula_volume | -0.65 | 5.22E-01 |
| CC_Posterior | -0.63 | 5.32E-01 |
| rh_inferiortemporal_volume | -0.61 | 5.43E-01 |
| rh_parahippocampal_volume | -0.60 | 5.48E-01 |
| lh_lateraloccipital_volume | -0.60 | 5.52E-01 |
| rh_insula_volume | -0.59 | 5.57E-01 |
| rh_entorhinal_volume | -0.59 | 5.61E-01 |
| rh_fusiform_volume | -0.58 | 5.67E-01 |
| rh_caudalanteriorcingulate_volume | -0.57 | 5.69E-01 |
| LeftAccumbensarea | -0.57 | 5.72E-01 |
| rh_rostralanteriorcingulate_volume | -0.57 | 5.73E-01 |
| LeftVentralDC | -0.56 | 5.76E-01 |
| 5thVentricle | 0.54 | 5.89E-01 |
| lh_lingual_volume | -0.54 | 5.91E-01 |
| lh_bankssts_volume | -0.53 | 5.98E-01 |
| LeftThalamusProper | 0.53 | 5.99E-01 |
| rh_pericalcarine_volume | -0.52 | 6.06E-01 |
| lh_pericalcarine_volume | -0.52 | 6.06E-01 |
| lh_cuneus_volume | -0.49 | 6.28E-01 |
| BrainStem | 0.48 | 6.33E-01 |
| RightThalamusProper | -0.47 | 6.37E-01 |
| LeftInfLatVent | 0.47 | 6.37E-01 |
| lh_medialorbitofrontal_volume | -0.44 | 6.65E-01 |
| rh_lingual_volume | -0.41 | 6.86E-01 |
| rh_precentral_volume | -0.40 | 6.88E-01 |
| lh_frontalpole_volume | -0.40 | 6.90E-01 |
| rh_superiorparietal_volume | -0.37 | 7.11E-01 |
| lh_rostralanteriorcingulate_volume | -0.36 | 7.21E-01 |
| rh_parsopercularis_volume | -0.35 | 7.27E-01 |
| RightHippocampus | -0.33 | 7.39E-01 |
| rh_medialorbitofrontal_volume | -0.33 | 7.41E-01 |
| lh_inferiortemporal_volume | -0.32 | 7.50E-01 |
| CC_Central | 0.32 | 7.54E-01 |
| rh_bankssts_volume | -0.30 | 7.66E-01 |
| lh_caudalanteriorcingulate_volume | -0.29 | 7.74E-01 |
| lh_postcentral_volume | -0.27 | 7.87E-01 |
| RightInfLatVent | -0.27 | 7.91E-01 |
| CC_Posterior | 0.26 | 7.94E-01 |
| lh_caudalmiddlefrontal_volume | -0.25 | 8.01E-01 |
| CorticalWhiteMatterVol | 0.25 | 8.02E-01 |
| lh_entorhinal_volume | 0.24 | 8.09E-01 |
| lh_temporalpole_volume | -0.23 | 8.17E-01 |
| 5thVentricle | 0.20 | 8.43E-01 |
| RightAmygdala | 0.19 | 8.47E-01 |
| rh_caudalmiddlefrontal_volume | 0.16 | 8.72E-01 |
| lh_transversetemporal_volume | -0.16 | 8.72E-01 |
| LeftCaudate | -0.15 | 8.80E-01 |
| rh_transversetemporal_volume | -0.14 | 8.88E-01 |
| RightVentralDC | -0.14 | 8.90E-01 |
| LeftCerebellumCortex | 0.12 | 9.09E-01 |
| RightPallidum | 0.11 | 9.14E-01 |
| lh_frontalpole_volume | -0.09 | 9.30E-01 |
| CC_Mid_Posterior | -0.09 | 9.32E-01 |
| RightCaudate | -0.07 | 9.42E-01 |
| lh_supramarginal_volume | -0.06 | 9.49E-01 |
| RightCerebellumCortex | -0.03 | 9.76E-01 |
| LeftAmygdala | 0.03 | 9.78E-01 |
| CC_Anterior | 0.02 | 9.84E-01 |
| LeftHippocampus | -0.02 | 9.85E-01 |
| lh_precentral_volume | 0.00 | 9.99E-01 |

## Supplementary Table 5

To investigate whether the observed effects were being driven by outliers, we removed the subjects whose ICV-corrected total gray matter volume was outside of two standard deviations of the grand mean and re-ran our analyses. One adult with DS and four adults with WS were removed. (a) Results from the analyses comparing the relationship between volume and age in DS vs. TD with the outliers removed are presented, which can be directly compared to results presented in Table 2. (b) Results from the analyses comparing the relationship between volume and age in DS vs. WS with the outliers removed are presented, which can be directly compared to results presented in Table 3. Bolded p-values passed Bonferroni correction.

### Supplementary Table 5a

| **ROI** | **Group (DS vs. TD) x Age Interaction** | | |
| --- | --- | --- | --- |
|  | **t** | **p-value** | **Difference in R^2^** |
| LILV | 2.39 | 1.8E-02 | 0.04 |
| RILV | 2.12 | 3.6E-02 | 0.05 |
| Left Superior Parietal | -3.64 | **4.0E-04** | 0.07 |
| Left Inferior Parietal | -3.48 | 7.1E-04 | 0.07 |
| Left Pars Orbitalis | -3.20 | 1.7E-03 | 0.06 |
| Right Post Central Gyrus | -3.26 | 1.4E-03 | 0.06 |

### Supplementary Table 5b

| **ROI** | **Group (DS vs. WS) x Age Interaction** | | |
| --- | --- | --- | --- |
|  | **t** | **p-value** | **Difference in R^2^** |
| Right Lateral Ventricle | 4.20 | **1.34E-04** | 0.024 |
| Left Lateral Ventricle | 3.73 | 5.50E-04 | 0.022 |

## Supplementary Figure 1

This figure shows results from post-hoc analyses which excluded subjects whose ICV-normalized gray matter volumes fell outside two standard deviations of the grand mean for their respective group (one with DS and four with WS). These plots can be directly compared to Figures 2a,b (for Suppl. Figure 1a,b) and 3a,b,c,d (for Suppl. Figure 1c,d,e,f) in the main manuscript. Regional brain volumes normalized to ICV are plotted in relationship to age across the three subject groups (Down syndrome (DS) in red, typical development (TD) in green, and William syndrome (WS) in blue) in (a) the left inferior lateral ventricle (LILV), (b) right inferior lateral ventricle (RILV), (c) left inferior parietal lobe, (d) left pars orbitalis, (e) left superior parietal lobe, and (f) right post central gyrus.

## Supplementary Figure 1
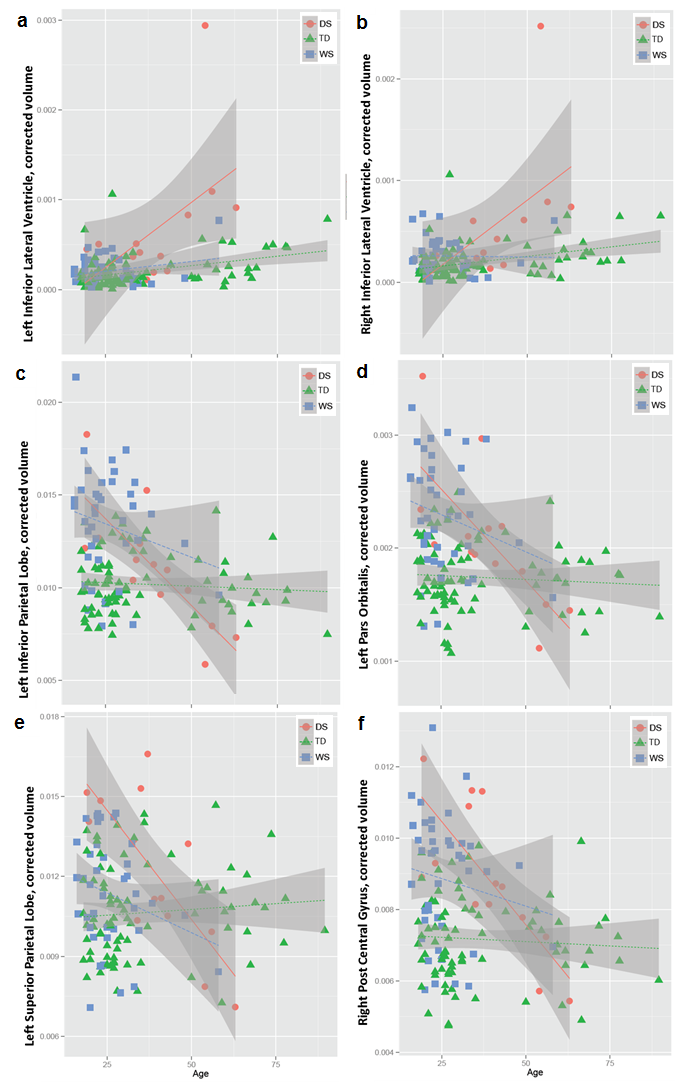


## Supplementary Figure 2

Regional brain volumes normalized to ICV are plotted in relationship to Dementia Questionnaire for People with Learning Disabilities (DLD) sum of cognitive scores (SCS) across the Down syndrome subject group in: (a) Right inferior lateral ventricle (RILV) and (b) Left inferior lateral ventricle (LILV). Subject data points are marked as *APOE* ε4 carriers (x) or non-carriers (o). For visualization purposes, we have overlaid a linear trend line for all data points, but note this line does not reflect covariate adjustments in the statistical model tested.

## Supplementary Figure 2

**
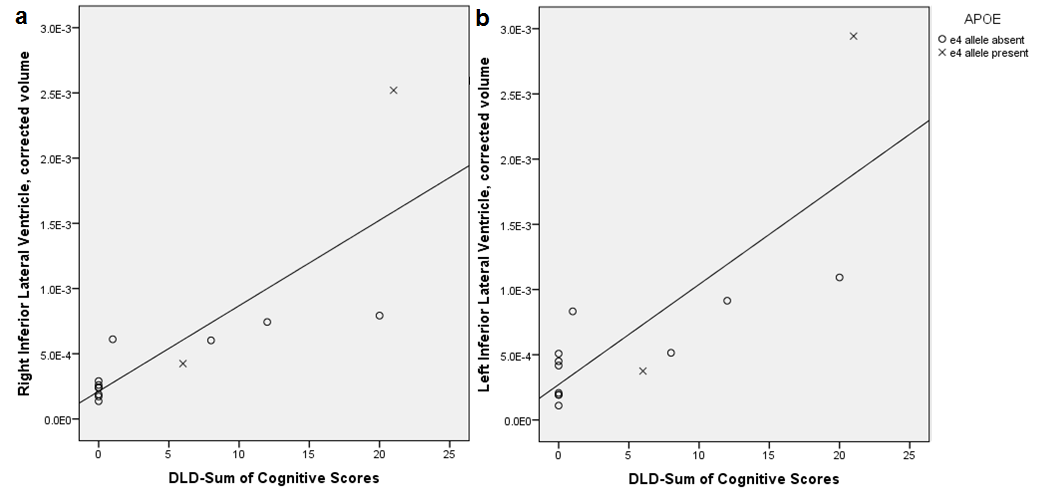
**

## Supplementary Figure 3

Regional brain volumes normalized to ICV are plotted in relationship to Dementia Questionnaire for People with Learning Disabilities (DLD) sum of social scores (SOS) across the Down syndrome subject group in: (a) Right inferior lateral ventricle (RILV) and (b) Left inferior lateral ventricle (LILV). Subject data points are marked as *APOE* ε4 carriers (x) or non-carriers (o). For visualization purposes, we have overlaid a linear trend line for all data points, but note this line does not reflect covariate adjustments in the statistical model tested.

## Supplementary Figure 3


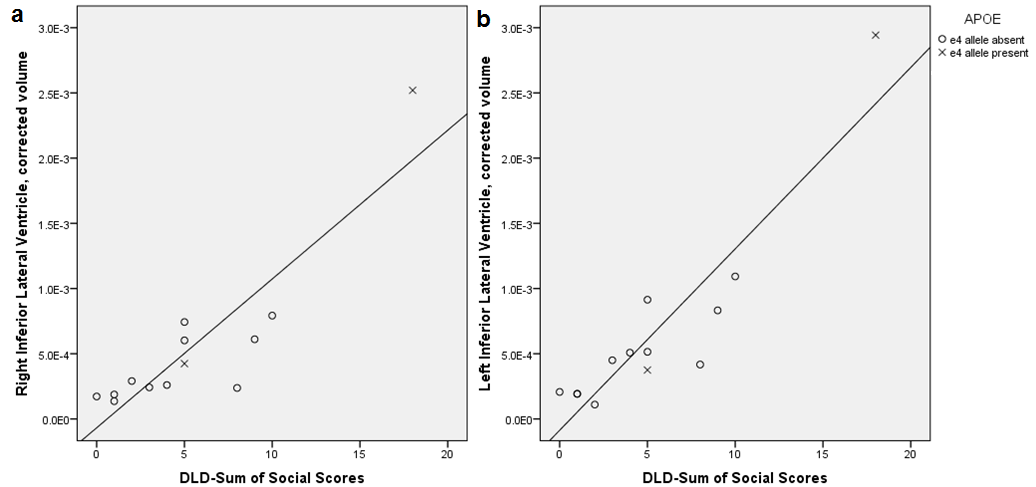


## Supplementary Figure 4

While *APOE* ε4 carrier status showed a trend for association with regional brain volume, a larger sample size will be needed to accurately estimate this effect. For the purpose of visualization, regional brain volumes normalized to ICV are plotted in relationship to age across the Down syndrome subject group, with adults marked as *APOE* ε4 carriers (x) or non-carriers (o) in: (a) Left inferior lateral ventricle (LILV) and (b) Right inferior lateral ventricle (RILV). For visualization purposes, we have overlaid a linear trend line for all data points, but note this line does not reflect covariate adjustments in the statistical model tested.

## Supplementary Figure 4


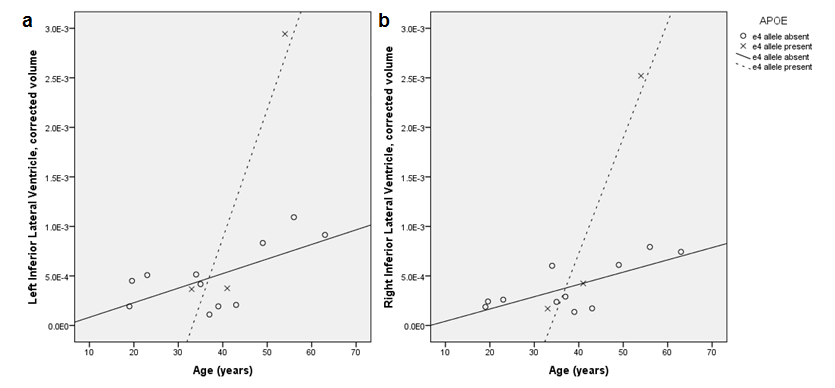

Supplement: Additional file 1: Table S1a — Regions of interest included in analysis. Table S1b. Regions of interest not included in analysis. Table S2. Additional demographics for Down syndrome participants, including APOE ϵ4 genotype, test scores for the Dementia Questionnaire for People with Learning Disabilities (DLD) (sum of cognitive scores (SCS) and sum of social scores (SOS)), and the Kaufman Brief Intelligence Test (KBIT) composite score. Table S3. complete results from analysis of relationship between volume and age comparing the Down syndrome (DS) and Williams syndrome (WS) groups to the typically developing (TD) controls. Table S4. Results from analysis comparing relationship between volume and age between participants with Down syndrome and Williams syndrome. Table S5. To investigate whether the observed effects were being driven by outliers, we removed the subjects whose ICV-corrected total gray matter volume was outside of two standard deviations of the grand mean and re-ran our analyses. Figure S1. This figure shows results from post hoc analyses which excluded subjects whose ICV-normalized gray matter volumes fell outside two standard deviations of the grand mean for their respective group (one with DS and four with WS). Figure S2. regional brain volumes normalized to ICV are plotted in relationship to Dementia Questionnaire for People with Learning Disabilities (DLD) sum of cognitive scores (SCS) across the Down syndrome subject group in: (a) right inferior lateral ventricle (RILV) and (b) left inferior lateral ventricle (LILV). Figure S3. Regional brain volumes normalized to ICV are plotted in relationship to Dementia Questionnaire for People with Learning Disabilities (DLD) sum of social scores (SOS) across the Down syndrome subject group in: (a) right inferior lateral ventricle (RILV) and (b) left inferior lateral ventricle (LILV). Figure S4. While APOE ϵ4 carrier status showed a trend for association with regional brain volume, a larger sample size will be needed to accurately estimate t [file 1866-1955-6-8-S1.docx]
